# Unraveling the landscape of non-melanoma skin cancer through single-cell RNA sequencing technology

**DOI:** 10.3389/fonc.2024.1500300

**Published:** 2024-11-04

**Authors:** Guorong Yan, Xiuli Wang, Guolong Zhang

**Affiliations:** ^1^ Department of Phototherapy, Shanghai Skin Disease Hospital, School of Medicine, Tongji University, Shanghai, China; ^2^ Skin Cancer Center, Shanghai Skin Disease Hospital, School of Medicine, Tongji University, Shanghai, China; ^3^ Institute of Photomedicine, School of Medicine, Tongji University, Shanghai, China

**Keywords:** non-melanoma skin cancer, single-cell RNA sequencing, scRNA-seq, intratumoral heterogeneity, drug resistance, recurrence

## Abstract

Non-melanoma skin cancer (NMSC) mainly includes basal cell carcinoma, cutaneous squamous cell carcinoma, and Merkel cell carcinoma, showing a low mortality rate but the highest incidence worldwide. In recent decades, research has focused on understanding the pathogenesis and clinical treatments of NMSC, leading to significant advances in our knowledge of these diseases and the development of novel therapies, including immunotherapy. Nevertheless, the low to moderate objective response rate, high recurrence, and therapeutic resistance remain persistent challenges, which are partly attributable to the intratumoral heterogeneity. This heterogeneity indicates that tumor cells, immune cells, and stromal cells in the tumor microenvironment can be reshaped to a series of phenotypic and transcriptional cell states that vary in invasiveness and treatment responsiveness. The advent of single-cell RNA sequencing (scRNA-seq) has enabled the comprehensive profiling of gene expression heterogeneity at the single-cell level, which has been applied to NMSC to quantify cell compositions, define states, understand tumor evolution, and discern drug resistance. In this review, we highlight the key findings, with a focus on intratumoral heterogeneity and the mechanism of drug resistance in NMSC, as revealed by scRNA-seq. Furthermore, we propose potential avenues for future research in NMSC using scRNA-seq.

## 1 Introduction

Non-melanoma skin cancer (NMSC) is the most prevalent malignant skin tumor, accounting for 99% of all skin cancers, with basal cell carcinoma (BCC) and cutaneous squamous cell carcinoma (cSCC) being the most two common types (1). Other less common types of NMSC encompass Merkel cell carcinoma (MCC), extramammary Paget’s disease (EMPD), and various skin adnexal carcinomas, including sebaceous carcinoma, apocrine adenocarcinoma, among others (2). The latest data shows that the worldwide incidence of new NMSC cases increased from 4.9 million in 2010 to 6.4 million in 2019, representing a 30.6% increase (3). This is attributable to a confluence of factors including increased life expectancy, heightened exposure to ultraviolet radiation (UVR), and advancements in data collection and diagnostic technologies (4). However, given the low mortality rate of most NMSCs and the accessibility of medical services in developing and underdeveloped countries (5), the global incidence of NMSC may be severely underestimated. Consequently, NMSC has now become a substantial economic burden (6).

With the development and innovation of next-generation sequencing (NGS) technologies, numerous dysregulated genes associated with their mutation, expression, and epigenetic modification during NMSC progression have been sought out (7–10). Bulk RNA-sequencing (RNA-seq) is a powerful tool for identifying tumor suppressor genes and oncogenes, showing advantages of low signal-to-noise ratio, and cost-effectiveness (11). Given that heterogeneity is the most defining feature of tumors (12), it is important to note that the limitations of this approach should be considered when applied to cancer biology, given its tissue-based nature. Firstly, bulk RNA-seq averages the gene expression across a pooled population of cells within the examined tissue, leading to a potential risk of masking the gene expression changes of rare cell populations. Furthermore, accurate cell composition cannot be obtained, despite the development of various deconvolution methods to estimate the cell composition from bulk RNA-seq data (13). The cell heterogeneity is evident not only in the variety of cell types and their respective states, but also in the dynamic plasticity during tumor progression and the drug resistance that develops after therapeutic interventions (14). Additionally, the evaluation of cell-to-cell complex crosstalk among the tumor microenvironment (TME) is also inaccessible by bulk RNA-seq. Fortunately, these difficulties are being increasingly resolved by the single-cell approach (15).

**Figure 1:**
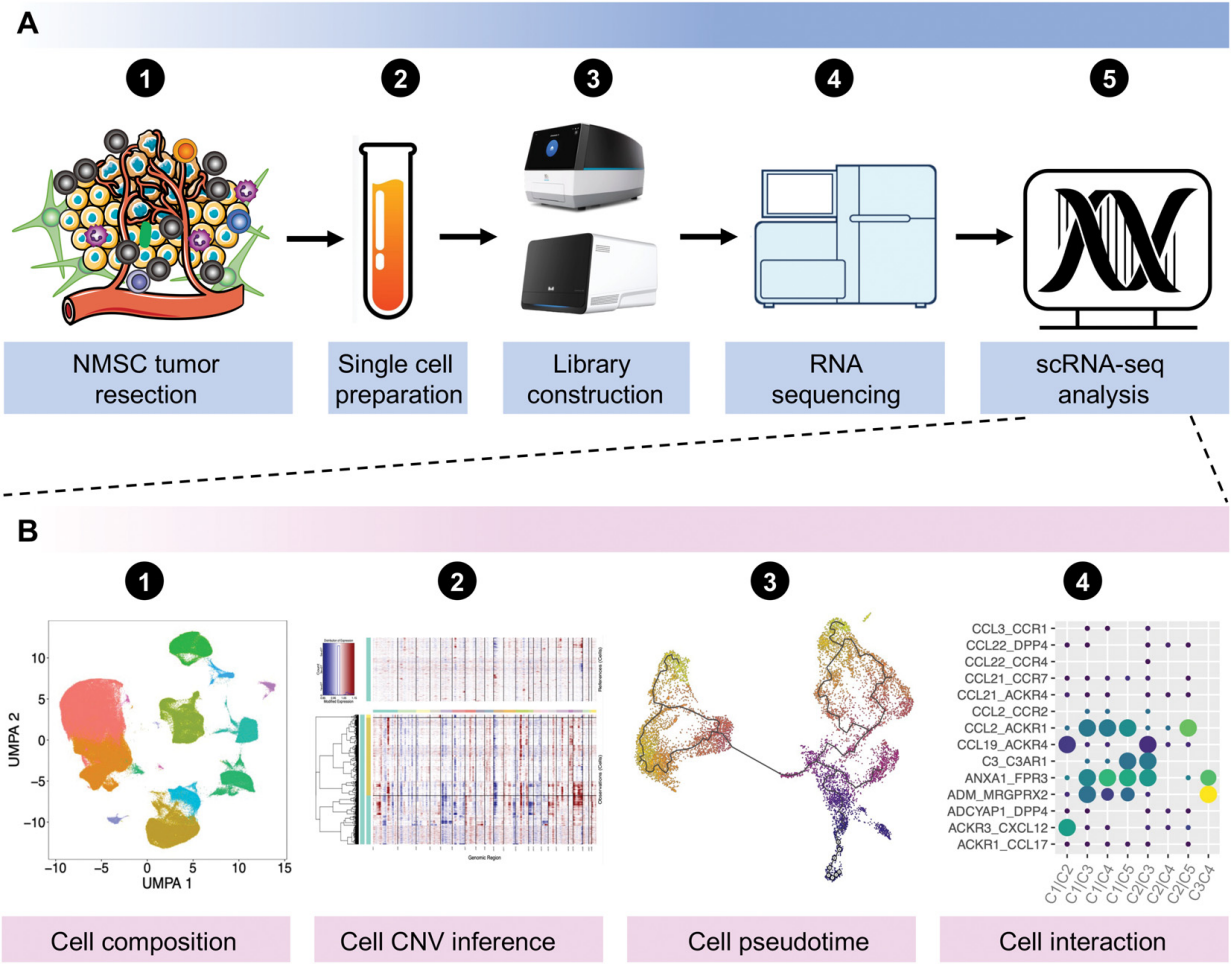
General workflow of scRNA-seq experiments from sequencing to data analysis. **(A)** General workflow of scRNA-seq experiments in NMSCs. **(B)** General analyses for scRNA-seq data. Data was downloaded and analyzed with the accession of GSE130973, GSE1993304, GSE144236, GSE200334, and GSE218170 from the Gene Expression Omnibus (GEO) database. NMSC, non-melanoma skin cancer; scRNA-seq, single-cell RNA sequencing; UMAP, uniform manifold approximation and projection; CNV, copy number variation.

Single-cell RNA sequencing (scRNA-seq) is a novel approach that enables high-throughput transcriptome sequencing at the single-cell level, effectively tackling the challenge of transcriptomic heterogeneity among cells within a sample (**Figure 1A**). The technology of scRNA-seq can be used for cell composition evaluation, copy number variation (CNV) inference of tumor cells, pseudotime analysis for different cell clusters, and cell communications (**Figure 1B**). Since Tang et al. published the first scRNA-seq technology in 2009 (15), the field has undergone rapid development and widespread application in the life sciences, including tumor biology (16), development (17), immunology (18), neuroscience (19), and drug development (20). However, while scRNA-seq has revolutionized our understanding of cellular heterogeneity and function, some limitations are not obviously neglectable. The technique is subject to technical biases, such as doublets or drop-out events where true gene expression levels are not captured, leading to incomplete and inaccurate datasets (21, 22). Additionally, the low throughput of some single-cell platforms can limit the number of cells that can be analyzed in a single experiment, potentially missing rare cell types. The high cost and complexity of the technology can also be barriers to its widespread adoption. Furthermore, the interpretation of single-cell data requires sophisticated bioinformatics tools and expertise, which may introduce errors or biases in the analysis (23). Therefore, the results explored from the scRNA-seq technology should be confirmed further by various biological experiment. Actually, some reviews have discussed the application and advancement of scRNA-seq in skin diseases (24, 25) and skin cancer (26, 27), with a particular focus on melanoma (28). Here, we primarily focused on the application of scRNA-seq in dissecting intratumoral heterogeneity, drug resistance, and the specific mechanism of cell-cell interactions in the progression of NMSC.

## 2 Classification of NMSC

Basal cell carcinoma (BCC) is the most common type of skin cancer, accounting for approximately 75% of all NMSC cases (29). BCC originates from epidermal cells, frequently occurs on the face, and some types of BCC, such as infiltrative BCC, often shows strong local invasiveness. The primary risk factor for BCC is UVR, with light-skinned individuals being more susceptible (30). Mutations in the Hedgehog (HH) signaling pathway (80%) and TP53 (60%) were predominantly found in BCC (31), and the aberrant activation of the HH signaling pathway has been proven to play a critical role in the majority of cases, making it a promising candidate for targeted therapies (32). However, only a 30–43% response rate was observed with HH signaling pathway inhibitors (Vismodegib or Sonidegib) (32) or PD-1 inhibitors (33), for metastatic BCC or with locally-advanced BCC, indicating a potential for acquired or intrinsic drug resistance (34, 35).

cSCC is the second most common NMSC, frequently occurring on the head and face of the elderly. Substantial evidence implicates that UVR and other non-UVR risk factors include immunosuppression, infections, specific drugs, and exogenous chemical mutagens involved in cSCC initiation (36). Furthermore, actinic keratosis (AK), as a precancerous lesion of cSCC, is closely related to the risk of cSCC. A cohort study involving more than 200,000 cases of AK showed that up to 1.92% of AK progressed to cSCC (37). Another multicenter clinical trial reported that the progression rate of severe AK (Olsen grade 3) to invasive cSCC was as high as 20.9% (38). Although the majority of cSCC can be treated by surgical resection or topically treated with imiquimod or 5-fluorouracil (39), a small proportion will progress into metastases with significant morbidity and mortality (40). Consequently, for multiple or locally advanced, metastatic cSCC, immune checkpoint inhibitors (ICI) can be applied, with an average objective response rate of 42–58% for anti-PD-1 antibodies (Cemiplimab, Pembrolizumab, Nivolumab) (41–44).

Merkel cell carcinoma (MCC) is a rare but highly aggressive neuroendocrine cancer of the skin, that has quadrupled in incidence over the past two decades (45). The development of MCC is linked to two primary factors: the integration of Merkel cell polyomavirus (MCPyV) into the host genome, which leads to the persistent expression of viral early genes, including small and large T antigens, or the mutagenic effects of UVR (46). Surgery remains the most common method by which primary MCC tumors are removed. For advanced MCC, Immunotherapy has now become the standard treatment, leading to a 31.8–68% objective response for different ICI agents (47, 48). In addition to BCC, cSCC, and MCC, there are several other less common types of NMSC, such as EMPD, sebaceous carcinoma, apocrine adenocarcinoma, and various other skin adnexal carcinomas (2).

## 3 Recent applications of scRNA-seq in NMSC

### 3.1 Basal cell carcinoma

BCC is the most common NMSC and numerous studies have demonstrated the existence of intra-tumoral heterogeneity (ITH) within BCC, encompassing tumor cells, immune cells, and stromal cells of BCC (49–52). For example, Huang et al. extensively depicted the dynamic cell heterogeneity among tumor epithelial cells, immune cells, myeloid cells, endothelial cells, and stromal cells of infiltrative BCC and revealed the inflammatory characteristics and CD4^+^ Treg-derived impaired immunity of BCC by scRNA-seq (49). Ganier and colleagues also conducted a comprehensive comparison of the cell composition differences between BCC healthy skin from different anatomical sites and found that there were significant expansions of *RGS5* ^+^ pericytes and *POSTN* ^+^ cancer-associated fibroblasts (CAFs) in BCC (52), the latter was in accord with the result of Chen et al. (49). Another prominent finding was that their data suggest that malignant cells of BCC could originate from cells of the inner and outer hair follicle bulb, given that they analyzed keratinocytes in the interfollicular epidermis and in follicular structures using scRNA-seq (52). However, due to differences in classification criteria or the selection of canonical marker genes, the heterogeneity of some cells cannot be compared across studies, such as fibroblasts (49, 51). In such instances, the sharing and integration of scRNA-seq data become of even greater importance.

The complicated cell-to-cell communication within the TME plays a pivotal role in regulating tumor progression. Through integrating single-cell and spatial transcriptomics, Yerly et al. uncovered the epithelial collective migration characteristics of tumor cells and extracellular matrix (ECM) remodeling features of CAFs from the invasive niche of invasive BCC, and Activin A likely promotes tumor cell collective migration by activating fibroblasts to remodel the ECM (50). Furthermore, this large degree of ECM remodeling in BCC is likely driven by the expression of collagen- and metalloproteinase-coding genes (51). Additionally, they also found tumor cells responded to the sudden burst of WNT5A-drived CAFs-specific inflammatory signaling pathways by producing heat shock proteins (51). In addition to the crosstalk between tumor cells and CAFs, a strong crosstalk between *ANGPT2* ^+^ lymphatic endothelial cells and leukocytes was observed, demonstrating a strong ability to promote leukocyte migration and activation of this lymphatic endothelial cell (49).

Advanced BCCs exhibit a high mutational burden and ITH, and typically occur drug resistance or relapse after Smoothened (SMO) inhibitors or ICI treatments (31, 53). Yao and colleagues employed scRNA-seq to determine the molecular characteristics of SMO inhibitor-resistant tumor cells, which showed high expression of *LY6D*, *LYPD3*, and *TACSTD2* (54). Mechanically, AP-1 and TGF-ß cooperativity drive the nuclear myocardin-related transcription factor (nMRTF) resistance pathway, which amplifies non-canonical GLI1 activity in the *LY6D* ^+^ resistant tumor cells (54). Indeed, the plasticity of tumor cells represents a significant contributing factor to drug resistance. BCC transdifferentiation into squamous cell carcinoma is a distinct and important form of resistance for BCC, discarding the dependency on the HH pathway (55). Interestingly, the *LY6D* ^+^ SMO inhibitor-resistant tumor cells, extensively cover the morphological and transcriptional cell states of basal to squamous cell carcinoma transition (56). With the transition processing, the expressions of *LYPD3*, *TACSTD2*, *LY6D*, *CD44*, and *MUC1* were increased, while the expression of GLI1 was decreased, indicating the occurrence of drug resistance (56, 57). As a supplement, the same research team also demonstrated the mechanism regarding LY6D^–^ tumor epithelial proliferation. A TREM2^+^ VCAM1^+^ macrophage population was identified and spatially and functionally defined by integrating the scRNA-seq, the co-detection by indexing (CODEX) multiplex system, and the cytometry by time of flight (CyTOF) approach, which was found to be adjacent to the highly proliferative LY6D^–^ lower tumor epithelium area and promoted LY6D^–^ tumor epithelial proliferation via secretion of the ligand oncostatin-M (OSM) (58). In addition to the resistance to the SMO inhibitor, Pich-Bavastro et al. also found that Activin A secreted by tumor cells would mediate the polarization of CAFs and macrophages, thereby facilitating the resistance of tumor cells to ICI by scRNA-seq (59).

Collectively, most studies focused on the infiltrative BCC and identified high heterogeneity within BCC, including tumor cells and immune cells, indicating the complexity of BCC (**Table 1**). For this reason, with the increase in the number of cells, a large number of cell subpopulations have been identified, but their exact functions in promoting or inhibiting cancer require further investigation. The investigation of drug resistance mechanisms in BCC is a highly active area of research, with notable progress having been made in understanding the role of LY6D^+/–^ tumor cells in BCC resistance (**Table 1**). However, the comparability of cell subpopulations across different studies needs to be further clarified, either by using identical marker genes, or the same classification criteria or by integrating and reanalyzing different datasets. Moreover, the multi-omics approach is gradually demonstrating its unique potential in deciphering the heterogeneity and drug resistance of BCC (52).

**Table 1:** Representative scRNA-seq studies in BCC.

Samples (n, donors)	Sequencing platforms	Key findings	Data accessions
Tumor samples from infiltrative BCC (n = 5) and peri-tumor skin samples (n = 3)	BD Rhapsody	Identified a strong crosstalk between *ANGPT2* ^+^ lymphatic endothelial cell and leukocytes	Be available upon request (49)
Tumor samples from infiltrative BCC (n = 5)	10× Genomics	uncovered epithelial collective migration features of tumor cells and ECM-remodeling features of CAFs	GSE181907 (50)
Tumor samples from BCC (n = 4) and peri-tumor skin samples (n = 2)	10× Genomics	Found tumor cells responded to the sudden burst of WNT5A-drived CAFs-specific inflammatory signaling pathways by producing heat shock proteins	GSE141526 (51)
Tumor and peri-tumor skin samples from the face of BCC (n = 8) and integrated with published normal skin samples	10× Genomics	Identified malignant cells of BCC could arise from cells of the inner and outer hair follicle bulb	E-MTAB-13085 (52)
Tumor samples from BCC (n = 4)	10× Genomics	Characterized the SMO inhibitor resistant tumor cells and the potential mechanism	Partial data from GSE141526 (54)
Tumor cells from treatment-naive BCC (n = 4) and resistant tumor cells from PD-1 treatment BCC (n = 11)	10× Genomics	Identified the transdifferentiation mechanism of BCC to SCC	Published data (56)
Tumor samples from BCC (n = 3) and merged some published BCC tumor samples	10× Genomics	Characterized the *LY6D* ^+^ tumor cells	phs003103.v1.p1 (57) and published data
Tumor samples from mouse BCC (n = 3) and integrated with some published human BCC samples	10× Genomics	Identified pro-tumorigenic *TREM2* ^+^ *VCAM1* ^+^ macrophage population	GSE204952 (58) and published data
Tumor samples from BCC (n = 5)	10× Genomics	Found Activin A secreted by tumor cells would mediate the polarization of CAFs and macrophages, facilitating ICI resistance	Published data (59)

### 3.2 Cutaneous squamous cell carcinoma

cSCC is the second most common NMSC after BCC. As the first scRNA-seq study in cSCC, Ji et al. extensively dissected the ITH of cSCC using 10 tumors and matched adjacent tissues, and identified a cluster of tumor-specific keratinocytes (TSKs) (60). The TSKs exhibited high expression of *MMP10*, *PTHLH*, *LAMC2*, and *SLITRK*, localizing to a fibrovascular niche at the leading edge of the tumor as their adjacent cells were enriched for CAFs and endothelial cells. Furthermore, they also exhibited an abundant epithelial-mesenchymal transition (EMT) signature which serves as a hub for intercellular communication with stromal and immune cells, contributing to cSCC progression, immunosuppression, and ITH. Compared with the 10× Genomics Chromium platform, the Smart-seq2 strategy shows the capacity to detect a greater number of genes within each cell. Based on this, Yan et al. obtained 350 single cells from six primary UV-induced cSCC and determined the gene expression and chromosomal copy number variation pattern in cSCC (61).

In addition to focusing on cSCC itself, the recurrence of cSCC also warrants attention. The treatment of recurrent cSCC is usually more challenging, therefore exploring the recurrence mechanisms of cSCC can help prevent its recurrence and facilitate its treatment. Recurrent cSCC trended to exhibit worse immunosuppressed microenvironment featured by T cell exclusion, CD8^+^ T cell exhaustion, and enrichment of pro-tumor *SPP1* ^+^ tumor-associated macrophages (TAMs) (62). Interestingly, a cluster of TSKs identified by Ji et al. in the primary cSCC (60), was also studied in the recurrent cSCC. Although the total number of cell communication events was reduced in the recurrent cSCC (possibly due to the smaller cell number in the recurrent cSCC), TSKs still demonstrated a robust communication with inflammatory CAFs (iCAFs) and *IL7R* ^+^ CAFs which may be associated with cSCC recurrence (62). Actually, *IL7R* ^+^ CAFs may be a subtype of iCAFs, as they expressed high levels of *IL6*, *CXCL1*, *CXCL8*, and other cytokines (62). Those studies highlight the ITH of cSCC and also emphasize the pivotal role of TSKs in cSCC progression and recurrence, through extensive communication with different kinds of CAFs.

cSCC can progress from normal skin under persistent UVR, or from AK, a precancerous lesion, or the *in situ* carcinoma, Bowen’s disease (BD). In recent years, there has been a growing body of research investigating this progression spectrum of cSCC, attempting to prevent and intercept at the early stages of cSCC. Generally, there was no significant difference in the proportion of different kinds of keratinocytes (63), or other immune and stromal cells between AK and normal skin (64), indicating a small change during the progression from normal skin to AK. Even so, some AK-specific keratinocytes or genes were still identified including *ALDH3A1*, *IGFBP2* from Zou et al. (63), and *IL1R2*, *WFDC2* from Li et al. (64). Similarly, frequent cell communications between AK-specific keratinocytes and papillary fibroblasts were observed in AK, especially through the *ANGPTL4*–*ITGA5* interaction, indicating the central role of the ANGPTL pathway in AK development (64). Additionally, the number of proliferative basal cells was found to be significantly increased in BD, compared with AK (63). During the progression of cSCC, malignant cells activate fibroblasts into tumor-promoting CAFs. Thus, Schütz et al. demonstrated the heterogeneity of CAFs between BD and cSCC (65) and found that iCAFs and myofibroblastic CAFs (myCAFs) were dominantly present in BD and cSCC, respectively, in contrast to their absence in AK. Further analyses showed that iCAFs may derive from pro-inflammatory FBs playing immunoregulatory function, while myCAFs may originate from mesenchymal FBs which are associated with ECM organization (65). However, although most studies emphasize the importance of different types of CAFs during cSCC progression and recurrence, It remains an unsolved mystery regarding fibroblasts transform into CAFs at which stage during the progression of cSCC, and through what mechanisms.

Immunosuppression is a key risk for cSCC. Frazzette et al. found that Immunocompromised organ transplant recipients exhibited reduced proportions of cytotoxic and naïve tumor-infiltrating lymphocytes through scRNA-seq, which may be the cause of increased cSCC risk (66). Immunotherapy has been widely used in the treatment of high-risk cSCC. Adoptive cytotoxic T cell transfer-based immunotherapy (ACT) has a good initial therapeutic effect on cSCC, but there is a high probability of recurrence. Using cSCC mouse models, Miao et al. found that tumor-initiating stem cells selectively express CD80, which inhibits T cell activity upon engagement with cytotoxic T lymphocyte antigen 4 (CTLA4), suggesting that blockade of these cells may repress cSCC recurrence (67). Additionally, using scRNA-seq and scTCR-seq, Yost et al. found that cSCC and BCC that respond to anti-PD-1 therapy are derived from their own characteristics, continuously recruiting new T cells, rather than reinvigorating pre-existing tumor-infiltrating T cells through scRNA-seq (68).

Taken together, similar to BCC, the heterogeneity was uncovered first in cSCC and highlighted the important role of TSKs in cSCC progression and recurrence (**Table 2**). Additionally, developing novel immunotherapeutic strategies that target the immunosuppressive microenvironment and prevent recurrence will be essential to improve patient outcomes. In recent years, an increasing number of studies have focused on the progression from normal skin to cSCC. The transition from AK to cSCC is marked by the activation of fibroblasts into tumor-promoting CAFs. However, the transformation of fibroblasts into CAFs during cSCC progression remains unclear.

**Table 2:** Representative scRNA-seq studies in cSCC.

Samples (n, donors)	Sequencing platforms	Key findings	Data accessions
Tumor and peri-tumor skin samples from cSCC (n = 10)	10× Genomics	Identified a population of TSKs in tumor	GSE144236 (60)
Tumor and peri-tumor skin samples from cSCC (n = 6), and healthy young skin (n = 3)	Smart-seq2	determined the gene expression and chromosomal copy number variation pattern	Be available upon request (61)
Tumor and peri-tumor skin samples from primary cSCC (n = 4), and Tumor samples from recurrent cSCC (n = 1)	BD Rhapsody	Uncovered the TME of Recurrent cSCC and identified cell-cell communications	Be available upon request (62)
AK, Tumor, and peri-tumor skin samples from cSCC (n = 6)	10× Genomics	Dissected the cell composition change from NS to AK, then to cSCC	GSE1993304 (63)
AK and peri-AK skin samples from AK (n = 3)	10× Genomics	Revealed AK-specific keratinocytes and their crosstalk with secretory-papillary fibroblasts	Be available upon request (64)
Tumor samples from cSCC (n = 5), tumor samples from BD (n = 2), sun-exposed skin samples (n = 3), and integrated some published data	10× Genomics	demonstrated the heterogeneity of CAFs between BD and cSCC	GSE218170 (65)
Tumor samples from immunocompetent cSCC (n = 5) and immune suppressed transplant patients with cSCC (n = 5)	10× Genomics	Dissected the TME of transplant recipients with cSCC and Decreased cytotoxic T cells in transplant patients with cSCC	GSE145328 (66)
Tumor samples from mouse cSCC model	Smart-seq2	Identified tumor-initiating stem cells selectively express CD80, which inhibits T cell activity	GSE108679 (67)
tumor samples cSCC (n = 4) post-ICI treatment	10× Genomics	Found clonal replacement of tumor-specific T cells after PD-1 treatment in cSCC	GSE123813 (68)

### 3.3 Merkel cell carcinoma

MCC is an aggressive skin cancer typically caused by the MCPyV and UVR. Similar to other NMSC, the prominent ITH was observed in MCC. Das et al. identified two distinct MCC tumor cell states: a “mesenchymal-like” state characterized by an inflammatory phenotype and a “well-differentiated neuroepithelial” state, which is associated with acquired resistance to ICIs (69). Additionally, the study demonstrated that manipulation of the intrinsic tumor cell into a “mesenchymal-like” state could lead to increased sensitivity to PI3K inhibitors, suggesting a potential non-genomic resistance mechanism (69). Furthermore, it seems that the heterogeneity of MCC cells was not due to their viral status, as one study reported the co-clustering of MCC cells from virus-positive and virus-negative tumor samples (70). Specifically, MCC cells can be classified into two subtypes according to the expression of neuroendocrine (NE) related genes, and *YAP1* suppresses cell-cycle progression in the MCC cells with high expression of NE genes in part through indirect downregulation of MCPyV large T antigen expression (70). The similarities and differences in gene signature between the “mesenchymal-like” and “high NE” states of MCC cells should be further investigated. Another study also reported the plasticity of MCC cells and they found that the inhibition of MCPyV T antigen expression could revert MCC cells to neuron-like cells revealing the potential conversion of an aggressive cancer phenotype to a differentiated state (71).

The immune cells and fibroblast states have also been dissected by scRNA-seq. Gherardin et al. identified a cluster of γδ T cells in MCC, exhibiting proinflammatory phenotype, which may be important effector cells and may serve as prognostic biomarkers and potential therapeutic targets for MCC (72). Previous studies have characterized tumor-specific T cell responses to MCPyV-positive MCC, which have typically been CD8^+^ T cells (73), but little is known about the T cell response to MCPyV-negative MCC. Therefore, Church et al. identified and characterized tumor-specific Th1-skewed CD4^+^ T cells targeting multiple neoantigens in a MCPyV-negative MCC patient who achieved a profound and durable partial response to anti-PD-L1 therapy (74). Fan et al. demonstrates that MCC-derived exosome-shuttle miR-375 induces fibroblast polarization by inhibiting *RBPJ* and *TP53* expression, leading to a pro-tumorigenic microenvironment (75).

Therapeutic resistance has been studied by many studies. MHC class I molecules (MHC-I), encoded by the human leukocyte antigen (HLA-A, -B, and -C) genes, are the primary molecules for presenting endogenous tumor antigens to CD8^+^ T cells. Genetic loss of a single HLA allele has been proven to be associated with ICI resistance in MCC (76), as it results in an inability to effectively present tumor antigens, preventing CD8^+^ T cells from recognizing and killing tumor cells. Intriguingly, Paulson et al. found that tumor cells evaded recognition by CD8^+^ T cells by selectively downregulating the expression of specific HLA genes, resulting in acquired adoptive cellular immunotherapy and ICI therapy resistance, through scRNA-seq in two metastatic MCC patients (77), while the histone deacetylase inhibitor *domatinostat* can reverse this effect by restoring HLA gene expression on MCC cells (78), and cause decreased cell viability and apoptosis through suppressing the transcription factor *HES1* (79). Meanwhile, the tumor-associated macrophages (TAMs) were the predominant myeloid subset in MCC, exhibiting an immunosuppressive gene signature, and were also associated with ICI resistance (80). Furthermore, Pulliam et al. found that the frequency of tumor-specific CD8^+^ T cells in the blood was positively correlated with ICI response and exhibited a senescent phenotype, while tumor-specific CD8^+^ T cells in the tumor showed an exhausted phenotype (73); and it was pointed out that the resistance to anti-PD-L1 originates from the decrease of HLA-I on tumor cells (73), which was consistent with the result of Paulson et al. (77). This suggests that ICI resistance at treatment re-initiation was not due to a shortage of tumor-specific T cells in the blood, but rather to the downregulation of HLA-I on tumor cells.

Overall, the ITH of MCC including MCPyV-positive and MCPyV-negative has been investigated through scRNA-seq technology (**Table 3**). At least two states of MCC tumor cells have been identified, and one of them is sensitive to therapeutic treatments. The reversion or conversion of different subtypes indicates the plasticity of MCC cells. Furthermore, the mechanism of ICI resistance should be paid more attention in the future.

**Table 3:** Representative scRNA-seq studies in MCC.

Samples (n, donors)	Sequencing platforms	Key findings	Data accessions
Tumor samples from treatment-naïve MCC (n = 9) and ICI resistant MCC (n = 2)	10× Genomics	Revealed the heterogeneity and plasticity of MCC tumor cell.	GSE226438 (69)
Tumor sample from MCPyV-positive (n = 6), -negative (n = 3) MCC	10× Genomics	Revealed the heterogeneity of MCC that were independent of MCPyV status	GSE189054 (70)
MCC cell line with or without T antigen knockdown	10× Genomics	Revealed the phenotypic reversion of MCC cells to neuron-like cells	GSE136867 (71)
FACS-sorted T cells in tumor sample from MCC (n = 1)	10× Genomics	Characterized γδ T cells	Supplementary data (72)
Matched pre- and post-PD-1 treatment tumor and PBMC samples from MCC (n = 8)	10× Genomics	Found cancer-specific T cells being more functional in blood than in tumor	GSE227054 (73)
FACS-sorted T cells in PBMC sample from MCPyV-negative MCC (n = 1)	Smart-seq2	Revealed T cell response to UV-induced neoantigens	Be available upon request (74)
CAFs from MCC (n = 3)	10× Genomics	Revealed miR-375 downregulates *RBPJ* and *TP53* to regulate fibroblast polarization	Figshare (75)
PBMC and tumor samples from metastatic MCC (n = 2) pre- and post- immunotherapy and ICI treatment	10× Genomics	Identified transcriptional suppression of the specific HLA genes presenting the targeted viral epitope in the resistant tumor cells	GSE117988 and GSE118056 (77)
MCC cell line treated with (n = 1) or without (n =1) domatinostat	10× Genomics	Revealed the effect of domatinostat in MCC	GSE141832 (78, 79)
Tumor sample from MCPyV-positive (n = 9) MCC	10× Genomics	Characterized TAMs and higher TAM levels were associated with ICI resistance	GSE227708 (80)

### 3.4 Other NMSC

EMPD is one rare type of NMSC, mainly localizing to the vulvar, perianal, scrotal, penile, and axillary regions. To date, only one scRNA-seq study has been performed in EMPD. Song et al. revealed significant epithelial and immune cell heterogeneity using tumor and peri-tumoral skin samples from one scrotal EMPD patient through scRNA-seq (81). Furthermore, this study found that *MSI1*, an RNA-binding protein, is overexpressed in the basal epithelial cells of human EMPD skin, and ectopic overexpression of *Msi1* in the epidermal basal layer of mice results in a phenotype resembling human EMPD at histopathological, single-cell, and molecular levels (81), which supports the *in situ* transformation theory, i.e. keratinocyte-to-Paget-like cell conversion of disease pathogenesis. Mechanically, *Msi1* activates the *Her2*-mTOR signaling pathway by directly suppressing *Cmtm5* and *Msi1* can also directly repress *Pten* to activate mTOR signaling. In conclusion, this study provides compelling evidence that the *Msi1*-mTOR signaling pathway plays a critical role in the pathogenesis of EMPD and that targeting mTOR signaling may offer a novel therapeutic approach for treating this disease. Although only one scRNA-seq study was carried out because of the rarity of EMPD, it is valuable to explore the recurrence mechanism of EMPD. Similarly, multi-omics at the single-cell level should be considered to uncover the pathogenies of EMPD.

## 4 Conclusion

**Figure 2:**
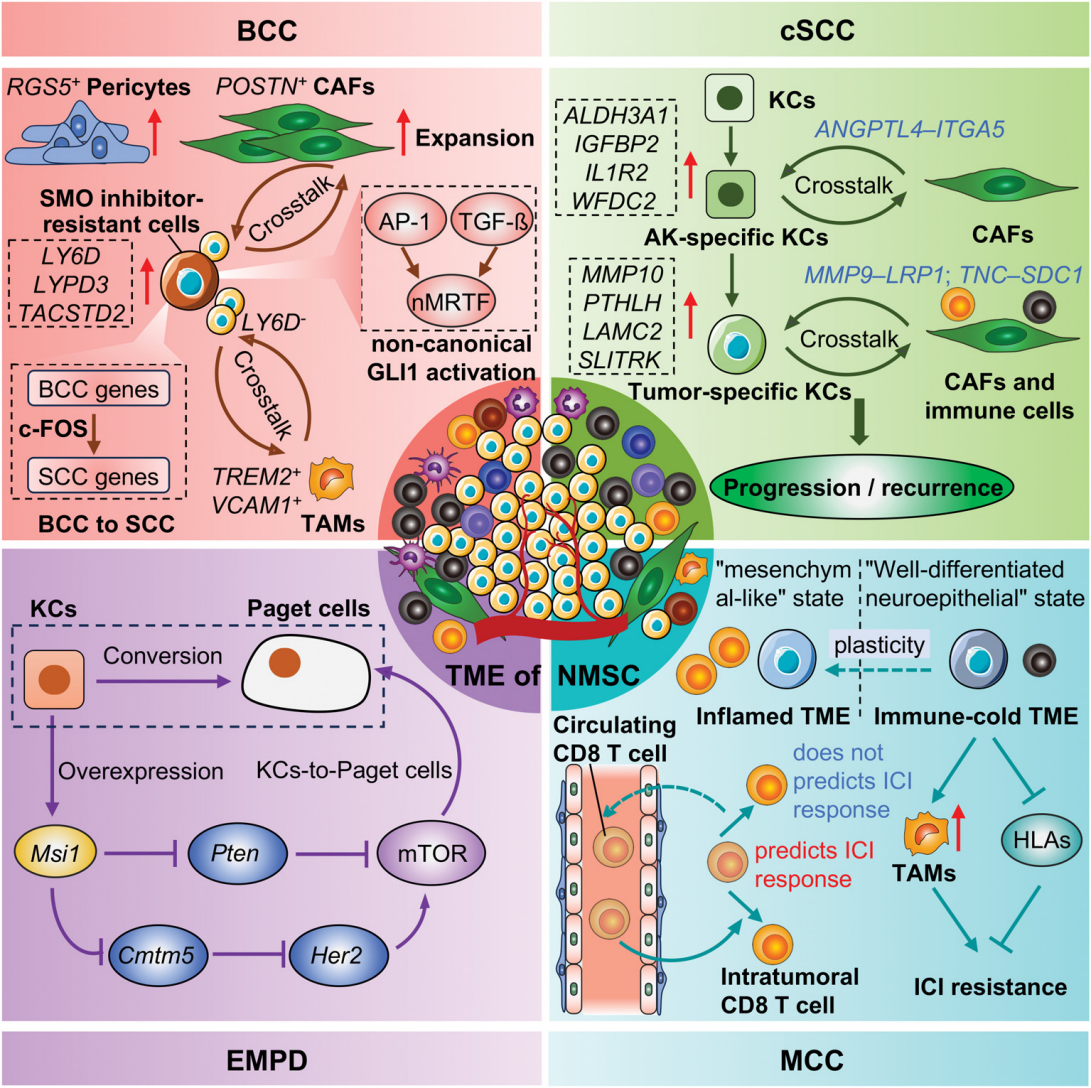
Representative advances of NMSC by scRNA-seq. KCs, Keratinocytes; CAFs, Cancer-associated fibroblasts; TAMs, tumor-associated macrophages; ICI, Immune checkpoint inhibitors; TME, Tumor microenvironment; NMSC, Non-melanoma skin cancer; BCC, Basal cell carcinoma; cSCC, Cutaneous squamous cell carcinoma; MCC, Merkel cell carcinoma; EMPD, Extramammary Paget’s disease.

A large number of scRNA-seq studies have been applied to NMSC (**Figure 2**), evolving from the initial revelation of cellular composition to the analysis of specific cellular subpopulations. The characterization of the diversity of functional states in tumor cells and immune cells of NMSC not only expanded our understanding of how tumors evolve and respond to ICI treatments, but also provided potential therapeutic targets to overcome treatment resistance. For example, identifying effectors that regulate BCC transdifferentiation into squamous cell carcinoma or mechanisms underlying tumor cell state transitions may present opportunities to reverse or prevent the emergence of treatment-resistant cell states. Regarding cSCC, The transition from AK to cSCC is marked by the activation of fibroblasts into CAFs, while this transformation during cSCC progression remains unclear. However, the incomplete transcriptomic information, the miss of rare cell types, sophisticated bioinformatics analyses, and the lack of spatial information also should be noted in scRNA-seq. Thus, Further research should consider some issues for scRNA-seq in NMSC. 1) More refined sampling methods of scRNA-seq or integrated spatial transcriptomic sequencing techniques are necessary to explore ITH in different tumor niches; 2) A unified naming system for cell subpopulations should be proposed to facilitate the comparison between similar studies conducted by different research teams, such as CAFs; 3) multi-omics approaches at single-cell level should be applied to comprehensively explore the pathogenesis, recurrence, and drug resistance in NMSC.
